# Supplementary material for: Different neuronal populations mediate inflammatory pain analgesia by exogenous and endogenous opioids
Source: eLife. 2020 Jun 10;9:e55289. doi: 10.7554/eLife.55289 (PMC7311172; doi:10.7554/eLife.55289)
Supplement: Transparent reporting form [file elife-55289-transrepform.docx]

***eLife’s* transparent reporting form**

We encourage authors to provide detailed information *within their submission* to facilitate the interpretation and replication of experiments. Authors can upload supporting documentation to indicate the use of appropriate reporting guidelines for health-related research (see [EQUATOR Network](http://www.equator-network.org/%20)), life science research (see the [BioSharing Information Resource](https://biosharing.org/)), or the [ARRIVE guidelines](http://www.plosbiology.org/article/info:doi/10.1371/journal.pbio.1000412) for reporting work involving animal research. Where applicable, authors should refer to any relevant reporting standards documents in this form.

If you have any questions, please consult our Journal Policies and/or contact us: [editorial@elifesciences.org](mailto:editorial@elifesciences.org).

**Sample-size estimation**

- You should state whether an appropriate sample size was computed when the study was being designed
- You should state the statistical method of sample size computation and any required assumptions
- If no explicit power analysis was used, you should describe how you decided what sample (replicate) size (number) to use

Please outline where this information can be found within the submission (e.g., sections or figure legends), or explain why this information doesn’t apply to your submission:

Sample sizes were determined according to experiments and field standards, and no statistical method was used to calculate the sample size. For the behavioural experiments, at least 5 mice for each group were used. For the data of *in situ* hybridization and immunohistochemistry, at least 3 mice were used in analysis. All the exact numbers of samples can be found in the figure legends.

**Replicates**

- You should report how often each experiment was performed
- You should include a definition of biological versus technical replication
- The data obtained should be provided and sufficient information should be provided to indicate the number of independent biological and/or technical replicates
- If you encountered any outliers, you should describe how these were handled
- Criteria for exclusion/inclusion of data should be clearly stated
- High-throughput sequence data should be uploaded before submission, with a private link for reviewers provided (these are available from both GEO and ArrayExpress)

Please outline where this information can be found within the submission (e.g., sections or figure legends), or explain why this information doesn’t apply to your submission:

For *in situ* hybridization and immunohistochemistry experiments, each mouse was considered as a biological replicate. For most of the behavioral experiments, data are collected from at least two batches of mice, except for the data of CFA-induced pain, frequency panels of von Frey test and Figure 7e-g, which only one batch of mice are tested in the experiments. To exclude the bias, experimenters are blinded to the grouping situation of mice. The exact number of the mouse can be found in the figure legend.

In Figure 7a-g, mice with improper fiber implantation sites or bad GCaMP6s expression are excluded from the data. In Figure 7h-o, mice with bad MOR re-expression in both sides of parabrachial nucleus are excluded from the data. No outliers were removed from the data.

**Statistical reporting**

- Statistical analysis methods should be described and justified
- Raw data should be presented in figures whenever informative to do so (typically when N per group is less than 10)
- For each experiment, you should identify the statistical tests used, exact values of N, definitions of center, methods of multiple test correction, and dispersion and precision measures (e.g., mean, median, SD, SEM, confidence intervals; and, for the major substantive results, a measure of effect size (e.g., Pearson's r, Cohen's d)
- Report exact p-values wherever possible alongside the summary statistics and 95% confidence intervals. These should be reported for all key questions and not only when the p-value is less than 0.05.

Please outline where this information can be found within the submission (e.g., sections or figure legends), or explain why this information doesn’t apply to your submission:

All the statistical analysis methods, exact p-values and N values can be found in the figure legends and the section of materials and methods. We have also plotted the individual data, mean and SEM in each figure.

(For large datasets, or papers with a very large number of statistical tests, you may upload a single table file with tests, Ns, etc., with reference to sections in the manuscript.)

**Group allocation**

- Indicate how samples were allocated into experimental groups (in the case of clinical studies, please specify allocation to treatment method); if randomization was used, please also state if restricted randomization was applied
- Indicate if masking was used during group allocation, data collection and/or data analysis

Please outline where this information can be found within the submission (e.g., sections or figure legends), or explain why this information doesn’t apply to your submission:

In most of our experiments, the groups of mice are decided by their genotypes. In the experiments of drug treatment or virus injection, mice are randomly assigned to each group.

**Additional data files (“source data”)**

- We encourage you to upload relevant additional data files, such as numerical data that are represented as a graph in a figure, or as a summary table
- Where provided, these should be in the most useful format, and they can be uploaded as “Source data” files linked to a main figure or table
- Include model definition files including the full list of parameters used
- Include code used for data analysis (e.g., R, MatLab)
- Avoid stating that data files are “available upon request”

Please indicate the figures or tables for which source data files have been provided:

All data are included in the figures and supplement files.
